# An Ethnobotanical Survey of Medicinal Plants Used in Papantla, Veracruz, Mexico

**DOI:** 10.3390/plants8080246

**Published:** 2019-07-24

**Authors:** Eduardo Alberto Lara Reimers, Eloy Fernández C., David J. Lara Reimers, Petra Chaloupkova, Juan Manuel Zepeda del Valle, Luigi Milella, Daniela Russo

**Affiliations:** 1 Department of Crop Sciences and Agroforestry, Faculty of Tropical AgriSciences, Czech University of Life Sciences Prague, Praha 6—Suchdol, Kamýcká 129, 165 00 Prague, Czech Republic; 2 Department of Forestry Engineering, Division of Forest Sciences, Autonomy University of Chapingo, Km 38.5 roadway, México–Texcoco, Chapingo, CP 56230, Mexico; 3 Department of Economics and Development, Faculty of Tropical AgriSciences, Czech University of Life Sciences Prague, Praha 6—Suchdol, Kamýcká 129, 165 00 Prague, Czech Republic; 4 Department of Regional Rural Development, Autonomy University of Chapingo Zacatecas, Zacatecas 98085, Mexico; 5 Department of Science, Basilicata University, V.le Ateneo Lucano 10, 85100 Potenza, Italy; 6 BioActiPlant s.r.l., Academic spinoff, Basilicata University, V.le Ateneo Lucano 10, 85100 Potenza, Italy

**Keywords:** ethnobotany, medicinal plants, fidelity level, informant’s consensus factor, use-reports

## Abstract

An ethnobotanical study was performed to collect information on the use of medicinal plants in Papantla, Veracruz, Mexico. The area has a high number of endemic species, and the social importance of the medicinal plants in the community is essential for public health and the conservation of traditional knowledge. This study identified the medicinal plants currently used, registered traditional knowledge, and documented the patterns of ailments treated in the indigenous communities of Totonacas. A total of 101 medicinal plants belonging to 51 families were described by 85 local informants. Asteraceae was the family with the highest number of plant species identified by these informants. Plant parts are used to treat several ailments, including venomous bites, gastro-intestinal disorders, infectious diseases and other disorders. Informants reported that the most common plant part used was the leaf tissue (55%), and they also took the herbal remedies orally (72%), and decoctions (38%) as well as infusions (29%) were the forms used to prepare these natural remedies. This study provides documentation of medicinal plants used in the Veracruz area of Mexico. Mexican people are still dependent upon medicinal plants, and in order to avoid their loss, certain measures of conservation for medicinal plants are needed.

## 1. Introduction

Traditional medicine is considered the first health care resource to treat ailments in several countries [1,2,3], and about 80% of people in the world depend upon traditional medicine, according to the World Health Organization (WHO) [4].

Traditional medicine studies include ethnomedicine, which involves the practices most used by people that live in rural areas and indigenous communities, and this ethnomedicine is affected by factors such as age, gender, economic activity, socio-economic level, migration, access to new health care systems, new herbal products and urbanisation [5,6,7]. The diversity of medicinal plants is very high in Mexico; the country contains a wide variety of plants (30,000) which have not been explored in their totality [8], and a significant number are endemic (uniquely native) species [9].

In Mexico, there are more than 50 indigenous languages spoken, and this country is very well-known for its biological diversity [10]. At the national level, the majority of Mexican indigenous populations live in rural areas (61.1% in communities with less than 2500 inhabitants).

The gathering and use of local resources are still important aspects of the phytotherapeutic traditions in many regions of Mexico. Plants are also used for ornamental, nutritional (food and fodder), pharmaceutical, aromatic, religious or construction purposes [9]. Aspects such as the richness and diversity of cultures will increase the relative importance and roles (uses) that each plant could have in respective communities. Mexican medicinal plants play an important role in public health among the local communities. In fact, traditional medicine is the first route to treat ailments, because many drugs are expensive, or are not always available locally [11].

Mexico has 31 states and a federal district, and Veracruz is one of the richest with regard to its biological and cultural diversity [12]. The medicinal flora of Veracruz has been used as remedies to treat several diseases, including those of the digestive system, skin and reproductive system, or for religious-cultural practices (often referred as “limpias”) [13]. In the Veracruz area, there are a number of indigenous groups who are keen consumers and practitioners of traditional medicine, such as the Totonacs, Tepehuas, Nahuas, Otomies, Popurcas, Zocos and the Popoluca Zaco, among others [14]. Previous studies carried out in the State of Veracruz registered more than 600 different taxa used for therapeutic purposes by the Popoluca (southern Veracruz) [15]. This supports the need to keep studying the areas previously studied in order to quantify and record these potential plants without antecedents. The wild flora in the state still play an important role, with different benefits for the social and ecological systems [16]. The Totonacs have a history of traditional medicine uses, and they are well-known for preserving a wide variety of plants. In the study area, as in other regions and countries, the recording of traditional knowledge is not widely promoted or supported, and the preservation of plant use knowledge is still carried out in oral form, and transmitted generation-by-generation [17]. Due to this fact, the aim of our work was to discover and document the traditional knowledge of medicinal plants in the 16 communities of Papantla, Veracruz, Mexico. A quantitative ethnobotanical approach was performed to analyse the medicinal use of plants and to select the important species in Papantla traditional medicine.

## 2. Results and Discussion

### 2.1. Characteristics of the Informants

There is a high prevalence in the use of medicinal plants and traditional knowledge in the Veracruz area. Table 1 provides the socio-demographic information such as residence, gender, age, occupation and annual expenditure in plants of the informants. Of the 85 indigenous people who answered the form, men represented the highest number (53%), and people from 70 to 85 years appeared to have a more extensive knowledge of these plants. In general, the gender was not significantly correlated to age and plant knowledge [18,19]. Even though all of the respondents generally used traditional plants, it has been common that, in many parts of the world, the women always demonstrate a more extensive knowledge in the use of plants [20]. This could be due to their roles in the family. 

Previous studies have shown that women usually have more knowledge about medicinal plants, and men know more about timber and handcraft species. This happens due to the sexual division of labour, and external factors resulting from the male migration of the young people inside the communities [18,21]. In our study, women on average cite two more plants and spend more money (17 plants, expenditure: $200) than their male counterparts (15 plants, expenditure: $176). Homemakers ($210 annually) generally spend more money for plants compared to those in other occupations; the lowest expenditure was observed for the sellers. The expenditure for plants during the year is equivalent to two days of work for the farmers. Case, et al. [22] mentions that local knowledge in plants increases with increasing geographical isolation; the people intensively use more plant species due to the scarcity of medical centres, specialists or medicines. Nevertheless, other studies have related family income (economic status) as the main factor in the number and the uses of known plants [23].

The keepers of the traditional knowledge were found to be the teachers (average of 22 plants), followed by farmers (18 plants), and housewives (15 plants). Academic staff (teachers) showed more knowledge of plants because they received children from different communities, and the social engagement in those schools promoted and applied different strategies to keep their costumes, languages and traditions alive inside the classroom.

In Mexico, the preservation of plant use knowledge is still carried out in oral form and transmitted generation by generation. In fact, informants declare that the origin of the traditional knowledge was given by the family (87%) and the specialist “shamans” (13%). More than half of the informants (58%) claimed to perceive a decrease (medium and high loss) in the traditional knowledge during their youth; the rest (42%) did not perceive any alarming decrease.

Seventy-eight of the informants (92%) were actively using medicinal plants in their daily lives, but 70 of these (82%) recur to the plants in the first instance to treat their ailments. Nevertheless, there were just 16% (14 people) going to specialists. The informants obtained the plants mostly from wild gathering (37%), the market (35%) and familiar gardens (28%). 

Some informants (29%) expressed that they do not have any expenditure in buying plants, since they know the surrounding areas where they can find them. The informants (38%) positively emphasised the usage of medicinal plants as part of their cultural uses (38%), and they consider them as an effective and cheap resource (35%) since their childhood to treat human disorders.

### 2.2. Mode of Preparation and Administration of Different Plant Parts

**Figure 1:**
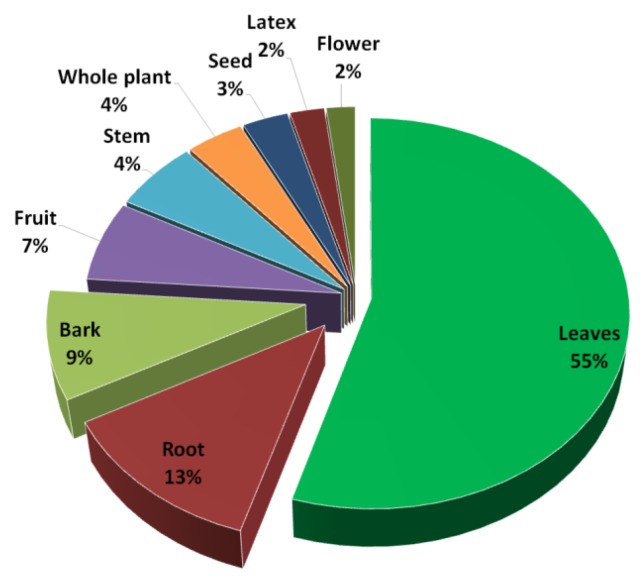
Plant parts used to prepare medicinal remedies.

Informants recognised 101 ethnobotanical plants belonging to 51 families distributed in 95 genera (Table 2), which were commonly used by most the of indigenous people for the treatments of 77 ailments. The most represented families were Asteraceae and Rutaceae, with eight plant species for each one, followed by Fabaceae (six species), Myrtaceae, Malvaceae and Apocynaceae (four species), and then Euphorbiaceae, Lamiaceae, Meliaceae and Poaceae with three species. Other families had two and one species each reported. Plant species of the Asteraceae family were the most used in Populoca, Veracruz [24], and not only in Papantla. This could be due to the abundance and wide variety of Asteraceae species in ecosystems in the northern and rainforest areas of Veracruz [16,18] and to their relative cultural importance. The plant list reported that a total of 61% of the species are native, 37% are exotic, and only 2% are endemic. The indigenous people keep using the local plants due to the great knowledge of the properties of these plants. The plant parts are usually consumed fresh (83%), and leaves (55%) represented the most common plant parts used by the informants to prepare their medicinal remedies. Leaves were followed by roots, bark, fruits, stems, whole plants, seeds, latex and flowers (Figure 1).

The main reason for the use of leaves was the ease of collecting them. Herbal medicines are either based on single species or mixed with other plant species. Nowadays, the use of the multi-treatment (contemporary and conventional medicine) becomes a usual method for people to save money, obtain more results, and reduce the allopathic effects from the modern medicaments [25]. The mixture of two or more plants is seen in this study; and it is known that the use of more than two herbs could contain a range of different active compounds and can modify its effect, enhancing or reducing the healing effect. If we considered each mixture as one single remedy [26], the list of natural remedies could be multiplied. Nonetheless, the toxic effects should be studied in depth. Many of the plants were used in minimal concentrations by the locals, but they are still unknown and unregistered. It is remarkable that the use of exotic plants has not been well investigated against normal symptoms.

**Figure 2:**
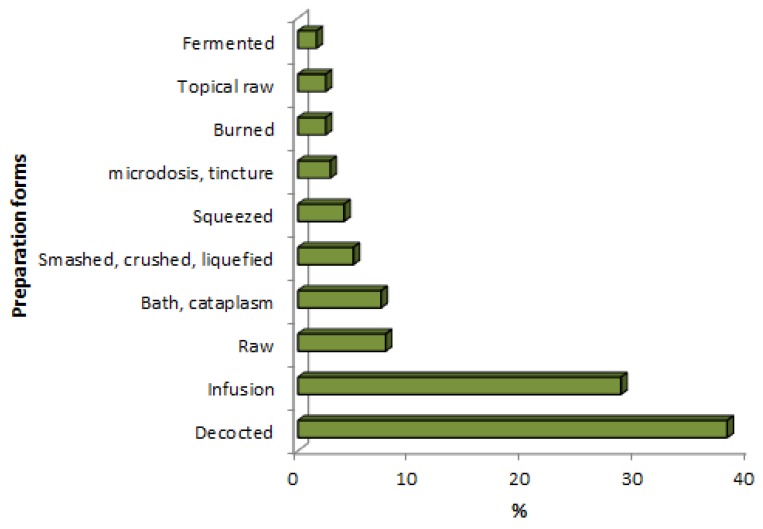
Remedies preparations expressed as percentages (%).

Two main routes of the administration of herbal remedies are reported: Oral (72%) and topical (28%) administration. Herbal remedies were prepared by using ten different methods. The main forms used were decoctions (38%) and infusions (29%), but raw plant material (11%) was eaten fresh in order to combat gastrointestinal disorders, for blood circulation and against local pain (tooth pain). Raw materials of leaves, whole plants or stems were used for rituals, such as bad wind and evil eye. Bath and cataplasm (7%) were applied to treat skin disorders (wounds) and to treat infective diseases such as chicken pox, smallpox and measles. Liquefied, crushed and smashed (5%) remedies were used fresh and extracted from different plant parts for the treatment of diabetes and kidney problems. Squeezed (4%), tinctured (3%) and burned (2%) remedies were mainly used to treat rheumatism, pain in the ears and wounds. The leaves of *H. patens* were used as therapy, where the leaves were burned and applied over the chest to promote breastfeeding in women. Bark, fruits and seeds were also fermented (2%) to prepare alcoholic drinks (Figure 2).

### 2.3. Use Reports, Informant’s Consensus Factor and Fidelity Level

Given the results of our observations, the studied communities had a significant variety of traditional uses, with a specific frame of ailments. A total of 77 ailments were grouped into 17 use-categories (Table 3) based upon the information gathered from those interviewed. 

The ICF was calculated for each ailment category, and the highest value was calculated for poisonous animal bites (ICF = 0.92), which was for the roots of two plant species, *Pentalinon andrieuxii* (Müll.Arg.) B. F. Hansen & Wunderlin (13 UR) and *Allium sativum* L. (1 UR), which were reported by informants to be used in tinctures for the treatment of snakebites. These species are the same species used to treat snakebite in Central America [27].

Problems related with different types of cancer (oncology use-category) showed an ICF of 0.91 with 5 species and 44 URs, followed by gastro-intestinal disorders (ICF = 0.89), with 29 species and 247 URs. Infective diseases and fever had an ICF of 0.87 with 13 species and 93 URs; kidney disorders and genital-urinary disorders reported a similar ICF, with 17 species associated with each. The use-category of liver disorders showed the lowest degree of consensus; only three informants mentioned three plant species to treat ailments belonging to this category (cirrhosis, hepatitis and liver disorders); probably informants had not exchanged their information. Malnutrition, poverty and environmental conditions are the main factors causing common ailments (digestive, respiratory and skin disorders), as previously reported [28,29]. This study also found that cancer (in the stomach, skin and gallbladder) and diabetes cases have increased recently, and this problem could be related to the diet in the region. Decoctions and infusions of leaves from *Asclepias curassavica* (16 URs), *Rauvolfia tetraphylla* (11 URs) and *Hamelia patens* (9 UR) were used to treat cancer, whereas plants, such as *Tecoma stans*, *Psidium guajava*, *Hamelia patens, Persea americana* and *Anacardium occidentale,* were included within the treatments for diabetes [30]. *Matricaria recutita*, *Mentha spicata*, *Psidium guajava*, and *Chenopodium ambrosioides*, are consumed in nine other Mexican states [5,31,32]. *Aloe vera*, *Piper auritum*, *Rutha chalepensis*, *Citrus limon*, *Annona reticulate*, and *Cocos nucifera* have been recorded to be widely used by indigenous people in central-southern Mexico [28,33].

The most commonly used species was *Hamelia patens* Jacq, with 77 URs. It is a large perennial shrub that has been used against a range of ailments by other indigenous communities in Mexico [34]. Totonacs use *Hamelia patens* to treat problems related to diabetes (UR = 18), gastrointestinal disorders (gastritis, colitis, and ulcers) (UR = 17), cancer (UR = 9), high blood pressure and blood circulation, respiratory problems, anaemia, breastfeeding, menstruation, skin disorders and wounds. Its medical effects has been proved in another countries, including India [35], in treatments of nervous shock for its antidepressant properties, athletes’ foot, skin lesions, insect bites, inflammation, rheumatism, headache, asthma and dysentery.

The leaves of *Persea americana* are commonly used by the Mexicans in infusions to treat gastrointestinal problems; nevertheless, previous reports have shown an increased use of the bark and seeds against diabetes, cholesterol and kidney problems in Central America [36,37]. Currently, *Aloe vera* plays an important role for its pharmacological effectiveness in treating a large number of ailments, such as skin problems, gastrointestinal problems, blood circulation problems, kidney problems and malnutrition, but it has been widely used by people with diabetes in Central America [30]. The use of *Aloe vera* has been spread throughout Latin America; and its ease of management and reproduction provided a cheap option for industry and people to grow it.

Additionally, the prevalence of new health problems that are present in tropical areas, such as dengue and Chikungunya, is getting more common around the world, and the way to combat them is a challenge for the people. The local people use coconut water as a strategy to reduce the impact of dehydration with the effects caused by Chikungunya. They also boil the mango leaves and mix them with the coconut water to drink and manage the fever caused by mosquito-borne diseases.

Our findings are in line with Alonso-Castro, et al. [29], where the main reasons of why people use medicinal plants in Mexico are related to their effectiveness and the low cost of usage and acquisition. However, they use traditional medicine as a complementary alternative to modern medicine, which is becoming more commonly used to treat diseases in Mexico in the past few decades [38].

The present study shows FL values varying from 23.4% to 100% (Table 2). The results reported 50 medicinal plant species having maximum 100% FL. The high FL shows the preference of these plant species by informants for the treatment of specific diseases [39].

## 3. Materials and Methods

### 3.1. Description of the Study Area

**Figure 3:**
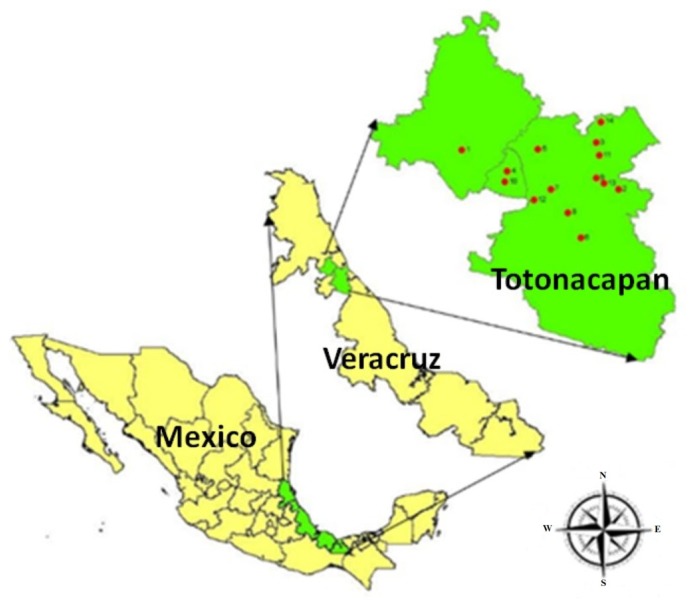
Study area of Veracruz, Mexico.

The study area, geographically known as Totonacapan, is located in the northern part of Veracruz (Figure 3). It is a part of the Northern Gulf Coastal Plain with an area of 4300 km^2^. This region represents about 5.97% of the total area of the state of Veracruz, and comprises 15 municipalities. The study area belongs to the municipality of Papantla, known as Papanteca, with coordinates 20°27′39″ S and 97°19′39″, W, and it lies at 180 m above sea level. It has an area of 1458.50 km^2^, which represents 2.03% of the state.

The climate is humid–warm, with an average annual temperature between 22 and 26 °C. The annual rainfall in the area varies between 1000 and 1500 mm. Totonacapan has a humid warm vegetation in most of its areas. The most common soils are Phaeozem-type Regosols and Vertisols, which are susceptible to erosion.

### 3.2. Socio-Economic Description

The municipal territory is mainly devoted to agriculture (68%), followed by 11% livestock, 13% housing; the remaining 8% are occupied by trade, public offices and public spaces. The total population in the Totonacapan amounts to 622,846 inhabitants, of which 204,934 people form the economically active population (32.9%). Nevertheless, it should be noted that 32.5% of Totonac’s population work in the primary sector (agriculture, fisheries and forestry), and around 66,000 people in Papantla have indigenous roots. According to Rivera and Ruiz-Ramírez [40], 77 of the 212 municipalities in the state have moderate poverty.

The ethnic composition of Veracruz is quite diverse and complex. Veracruz is the third-highest state in terms of the number of indigenous people (1,037,424) in México (CDI: National Commission for the Development of Indigenous Peoples) [41]. The state is divided into seven ethnic regions, and ethnic linguistic groups are located in the state of Veracruz. These groups still speak 14 different languages [Huasteco (Tenek), Popoluca, Mixe, Zoque, Chinanteco, Zapoteco, Mazateco, Mixteco, Otomí, Totonaca, Tepehua, Náhuatl from the Huasteca, Náhuatl from the Sierra de Zongolica, and Nahua from the south]. The most representative groups are Mazatecos, Totonacos and Zapotecos [40].

The Totonacos group lives in the city of Papantla and the surrounding areas; the tourism in this area helps them to sell handcrafts. Moreover, the performances of the sky dancers (Papantla flyers), and shamans are the main reason for tourism throughout the Mexican territory. It also has a remarkably high number of tourists who travel from different parts of the country to visit the shamans and buy the medicinal plants. The agriculture, livestock, forestry, traditional textile clothes, plants, fruits, practicing of traditional medicine and informal employment are the base of the local economy. Corn, beans, coffee, vanilla, bananas, citrons and oranges are the main crops produced in the region [7,18,42].

Previous studies performed in neighbour municipalities [18] have shown the number of farmers depending on the farm products, and discuss migration and a lack of good opportunities causing a loss of interest to preserve the traditional knowledge in the new generation. The study area is highly interesting, and represents a special combination of different factors to study the patterns of traditional medicine.

### 3.3. Ethnobotanical Analysis

Fieldwork was carried out from March to August of 2017 in 16 communities in the Papantla region (Adolfo López, Arroyo Grande, Carrizal, Cedros, Lahuas, Natividad, Panti, Papantla, Polutla, Poza Rica, Pozo Verde, San Antonio Xital, Veracruz, Spoupat, Volador and Zapotal). Before starting the survey, ethical approval for the study was first obtained from the indigenous organised group “Consejo de Ancianos de la Sabiduria Ancestral”. Likewise, the president of the indigenous group, Miss María Luisa Santes Santes, supported and accompanied the visits to the people interviewed; this was in order to explain to them the investigation’s purpose and to ask for their participation in this study. The questionnaires were supplied in the Spanish language and translated into the local language (Totonaco dialect) for the indigenous people who did not speak Spanish. Two translators accompanied the interviewer. Ethnobotanical information was collected from local inhabitants by using semi-structured questionnaires. A total of 85 informants were selected by snowball sampling. The informants were interviewed in their houses, in the streets and in local markets. Participant observation was also part of the interview, in order to have a better interpretation and analysis of the data reported by these informants.

The informants were asked to provide knowledge about the plant uses (local names, indication of use, used plant parts, places/methods/rituals of harvesting and administration mode). The informants were asked to show the place where they usually collect the plants. Many visits were conducted with the interviewed informants in order to collect, press and identify the medicinal plants. The plant material was collected by the authors and taxonomically identified. The botanical names of the species were verified with The Plant List (2013) (http://www.theplantlist.org) and voucher specimens were deposited in the Herbarium at the Chapingo Autonomous University.

### 3.4. Data Analysis

#### 3.4.1. Use Categories

Based on the information obtained from the indigenous people in the study area, all of the reported ailments were categorised into use-categories. The basic structure of ethnobotanical information is the use-report (UR) and this can be considered as an individual report of a specific taxon/drug for a certain use-category. When a plant is cited as “used”, it is considered as one “use-report”, but if one informant mentions the same plant to treat more diseases in the same category, it is considered as a single use-report.

#### 3.4.2. Informants’ Consensus Factor (ICF)

In this study, the level of homogeneity among the information collected from diverse informants for plant species in treating particular diseases was calculated by the informants’ consensus factor (ICF). It was estimated using the following formula [43]:

$$ICF=\frac{Nur-Nt}{Nur-1}$$

where, *Nur* is the number of UR in each disease category and *Nt* is number of species used. Low *ICF* values suggest that the plant is used randomly, or information on its use is not exchanged among informants, whereas a high exchange of information and a well-defined selection criterion in the community is reported for a plant with high ICF values.

#### 3.4.3. Fidelity level (FL)

Fidelity level (FL) represents the percentage of informants claiming the use of a certain plant for the same major purpose, which can be calculated for the most frequently reported diseases or ailments as:

FL (%) = (Np/N) × 100

where “Np” is the number of informants that claim a use of a plant species to treat a particular ailment, and “N” is the number of informants that use the plants as a medicine to treat any given disease or category [44].

## 4. Conclusions

The collected information indicates that the study area is rich in medicinal plants, and the results contribute to spread their uses. The social importance of the medicinal plants in the community is quite important for the public health and the conservation of traditional knowledge, and good management is required. In Papantla (Veracruz, Mexico), the indigenous population still depends upon medicinal plants to treat several ailments. The plants used have a mostly native origin. The species most frequently mentioned by the informants was *Hamelia patens* followed by *Persea americana*, *Bursera simaruba*, *Matricaria chamomilla*, *Mentha spicata* and *Aloe vera.* The safety and efficacy of cited plants needs to be investigated by phytochemical and pharmacological analysis, as it has been previously performed on several other traditionally used plant species [45,46,47].

## Figures and Tables

**Table 1:** Socio-demographic characteristics of the 85 informants.

Background Characteristic		No	%	Annual Expenditure (Mexican Pesos, $)
Residence	Rural	26	31	160
	Urban	59	69	200
Gender	Female	40	47	174
	Male	45	53	200
Age	>20	2	2	200
	21–30	13	15	233
	31–40	12	14	183
	41–50	16	19	173
	51–60	20	24	210
	61–70	14	17	120
	71–85	8	9	225
Occupation	Farmer	9	11	114
	Housewife	21	25	210
	Seller	4	5	50
	Teacher	2	2	200
	Worker	40	47	181
	Other	9	10	222

**Table 2:** Traditional use of medicinal plants among herbal practitioners in the study area (Totonac indigenous group).

Family	Scientific Name	Voucher Specimen	Common Name	Origin	Plant Part Used	Ailments	Category	Preparation Mode	Detailed Administration	UR	FL (%)
Amaranthaceae	*Beta vulgaris* L.	PA-01	betabel	Ex	root	**intestinal worms**	**G**	fresh	raw	1	100.0
						**stomach ache**	**G**	fresh	infusion	1	
Amaryllidaceae	*Allium cepa* L.	PA-02	cebolla morada	Ex	root	erection	D	fresh	crushed	1	66.7
						kidney problem	C	fresh	crushed	1	
						**veterinary fever in chicken**	**R**	fresh	smashed	4	
Amaryllidaceae	*Allium sativum* L.	PA-03	ajo	Ex	root	blood circulation	B	fresh	infusion	3	26.3
						cold	A	fresh	infusion	2	
						grains in the skin	O	fresh	bath	2	
			ajo con alcohol		root	liver problems	N	fresh	microdosis	1	
						**rheumatism**	**E**	fresh	tincture	5	
						snake bites	M	fresh	tincture	1	
Amaryllidaceae	*Allium sativum* L.		ajo con (with) aguacate		leaves	stomach ache	G	fresh	infusion	3	
						tooth pain	L	fresh	raw	2	
Anacardiaceae	*Spondias mombin* L.	PA-04	caña con jobo, durazno y piña	N	bark	alcoholic drink	R	dry	fermented	1	66.7
						**flu**	**A**	dry	decocted	6	
						tooth pain	L	dry	decocted	2	
Annonaceae	*Annona glabra* L.	PA-05	anona	N	leaves	diarrhoea	**G**	fresh	infusion	7	57.9
					fruit	drink	S	fresh		3	
					leaves	**stomach ache**	**G**	fresh	infusion	4	
					fruit	to have children	D	dry	squeezed	3	
					leaves	to have children	D	fresh	infusion	2	
Annonaceae	*Mangifera indica* L	PA-06	mango	Ex	seed	diarrhoea	**G**	fresh	decocted	4	100.0
Apiaceae	Arracacia atropurpúrea	PA-07	comino	N	leaves	diarrhoea	**G**	dry	decocted	6	100.0
Apiáceas	*Annona muricata*	PA-08	guanabana	N	leaves	cancer	I	fresh	infusion	7	34.4
					leaves	diabetes	H	fresh	infusion	6	
					leaves	**high pressure**	**B**	fresh	infusion	11	
Apiáceas	*Apium graveolens* L.		apio	Ex	stem	**cholesterol**	**B**	fresh	liquefied	8	100.0
Apocynaceae	*Pentalinon andrieuxii* (Müll.Arg.) B.F.Hansen & Wunderlin	PA-09	guaco enredadera	N	root	snake bites	M	dry	tincture	13	100.0
Apocynaceae	*Rauvolfia tetraphylla* L.	PA-10	cancerina	N	leaves	cancer	I	fresh	infusion	11	100.0
Arecaceae	*Cocos nucifera* L.	PA-11	coco and caña morada	Ex	leaves	blood circulation	B	fresh	raw	3	68.8
						**chinkunguya**	**P**	fresh	raw	5	
						**dengue**	**P**	fresh	raw	6	
					bark	stop bleeding in the parthum	D	fresh	decocted	2	
Arecáceas	*Acrocomia aculeata* (Jacq.) Lodd. ex Mart.	PA-12	coyol redondo palma	N	root	**diabetes**	**H**	dry	infusion	3	50.0
					bark	**eyes problem**	**Q**	dry	decocted	3	50.0
Asclepiadaceae	*Asclepias curassavica* L.	PA-13	hierva del sapo	N	leaves	**cancer**	**I**	fresh	decocted	16	59.,3
					leaves	diabetes	H	dry	infusion	5	
					leaves	kidney problem	C	fresh	decocted	6	
Asteraceae	*Artemisia ludoviciana* Nutt.	PA-14	estafiate	N	stem	**cholesterol**	**B**	fresh	infusion	12	100.0
Asteraceae	*Calea ternifolia* Oliv. ex Thurn	PA-15	zacate chichi	N	leaves, stem, flower	bile	G	fresh	infusion	5	70.6
						**diabetes**	**H**	fresh	infusion	12	
Asteraceae	*Cyclolepis genistoides* D.Don	PA-16	palo azul	Ex	bark	**kidney problem**	**C**	dry	decocted	4	100.0
Asteraceae	*Gnaphalium viscosum* Kunth	PA-17	gordolobo	N	whole plant	**cough**	**A**	fresh	infusion	2	100.0
Asteraceae	*Matricaria chamomilla* L.	PA-18	manzanilla	Ex	whole plant	**colic pain**	**D**	fresh	decocted	16	47.1
					leaves	eyes problem	Q	fresh	bath	4	
					whole plant	stomach ache	G	fresh	decocted	14	
Asteraceae	*Parthenium hysterophorus* L.	PA-19	chuchullate o tres hojitas	N	stem	anaemia	B	fresh	decocted	1	53.8
					leaves	**diabetes**	**H**	fresh	infusion	7	
					leaves	wounds	O	fresh	bath	2	
						wounds	O	fresh	decocted	3	
Asteraceae	*Tagetes erecta* L.	PA-20	flor de muerto	N	root	**stomach ache**	**G**	dry	decocted	6	100.0
Asteraceae	*Verbesina persicifolia* D.C	PA-21	huichin	N	leaves	**diabetes**	**H**	fresh	infusion	9	39.1
					leaves	gastritis	G	fresh	decocted	1	
					root	**high pressure**	**B**	fresh	decocted	9	39.1
					root	inflammation	E	fresh	bath	4	
Bignoniaceae	*Parmentiera aculeata* (Kunth) Seem.	PA-22	chote, chiote	N	flower	veterinary uses	R	fresh	decocted	7	50.0
						**kidney problem**	**C**	fresh	decocted	7	
Bignoniaceae	*Tecoma stans* (L.) Juss. ex Kunth	PA-23	tronadora (hoja de san pedro)	N	leaves	**infection in skin**	**O**	fresh	burned	2	100.0
Brassicaceae	*Nasturtium officinale* R. Br.	PA-24	berros	N	leaves	**anaemia**	**B**	fresh	bath	3	100.0
Burseraceae	*Bursera simaruba* (L.) Sarg.	PA-25	chaca		leaves	**fever**	**P**	fresh	cataplasm	45	100.0
Cactaceae	*Opuntia ficus-indica* (L.) Mill.	PA-26	Nopal, pepino and cascara	N	leaves	cholesterol	B	fresh	liquefied	2	66.7
						**clean stomach**	**G**	fresh	liquefied	8	
						diabetes	H	fresh	decocted	2	
Cannabacea	*Cannabis sativa* L.	PA-27	marihuana	Ex	whole plant	**rheumatism**	**E**	fresh	tincture	9	100.0
Cannabaceae	*Trema micrantha* (L) Blume	PA-28	puam	N	leaves	**chicken pox**	**P**	fresh	bath	8	100.0
						**measles**	**P**	fresh	bath	6	
Caricaceae	*Carica papaya L.*	PA-29	papaya		stem	**pain in ears**	**Q**	fresh	burned	5	100.0
Chenopodiaceae	*Chenopodium ambrosioides*	PA-30	epasote	N	leaves	**intestinal worms**	**G**	fresh	infusion	13	100.0
						**stomach ache**	**G**	fresh	infusion	12	
Commelinaceae	*Tradescantia spathacea* Sw.	PA-31	barquilla, maguey morado	N	leaves	grains in the mouth	L	fresh	squeezed	3	38.7
						kidney problem	C	fresh	infusion	8	
						respiratory system	A	fresh	infusion	8	
						**wounds**	**O**	fresh	burned	1	
						**skin infection**	**O**	fresh	bath	3	
						**wounds and bruises**	**O**	fresh	cataplasm	8	
Cucurbitaceae	*Cucurbita pepo* L.	PA-32	calabaza	N	latex	**scratches, wounds**	**O**	fresh	squeezed	4	100.0
Cucurbitaceae	*Sechium edule* (Jacq.) Sw.	PA-33	chayote	N	fruit	**cholesterol**	**B**	fresh	decocted	12	100.0
Euphorbiaceae	*Cnidoscolus chayamansa* Mc Vaugh	PA-34	chaya	N	leaves	**high pressure**	**B**	fresh	decocted	7	100.0
Euphorbiaceae	*Cnidoscolus tubulosus* (Müll.Arg.) I.M.Johnst.	PA-35	hortiga macho con espina	N	root	**kidney problem**	**C**	dry	decocted	5	61.1
					stem	**kidney problem**	**C**	fresh	infusion	6	
					latex	tooth pain	L	fresh	raw	7	
Euphorbiaceae	*Euphorbia hirta* L.	PA-36	riñonina	N	leaves	**kidney problem**	**C**	fresh	infusion	6	100.0
Euphorbiaceae	*Jatropha curcas* L.	PA-37	piñon	N	latex	**bleeding of the gums,**	**L**	fresh	topical raw	4	57.1
					leaves	grains	O	fresh	bath	1	
					latex	herpes	P	fresh	topical raw	2	
Fabaceae	*Bauhinia divaricata* L.	PA-38	pata de vaca	N	leaves	**diabetes**	**H**	fresh	infusion	4	44.4
						diarrhoea	G	fresh	infusion	3	
			Mixed with crushed rice		whole plant	dysentery	G	fresh	decocted	1	
					leaves	grains in the skin	O	fresh	bath	1	
Fabaceae	*Cassia fistula* L.	PA-39	hojasen	Ex	leaves	**colitis**	**G**	fresh	infusion	3	100.0
Fabaceae	*Erythrina caribaea* Krukoff & Barneby	PA-40	pichoco	N	bark	**push delivering in parthum**	**D**	fresh	decocted	2	100.0
Fabaceae	*Eysenhardtia polystachya* (Ortega) Sarg	PA-41	tarai (palo azul)	N	bark	**kidney problem**	C	dry	infusion	5	100.0
Fabaceae	*Gliricidia sepium* (Jacq.) Walp	PA-42	cocohuite	N	leaves	**fever**	P	fresh	tincture	2	100.0
Fabaceae	*Leucaena leucocephala* (Lam.) de Wit	PA-43	guaje	N	fruit	**intestinal worms**	G	fresh	raw	4	100.0
Geraniaceae	*Pelargonium* spp.	PA-44	malva con hortiga	Ex	leaves	chicken pox	P	fresh	decocted	1	50.0
						**grains**	**O**	fresh	decocted	1	
Lamiaceae	*Mentha* spicata L.	PA-45	hierva buena	Ex	leaves	colic pain	D	fresh	infusion	14	57.6
						**stomach ache**	**G**	fresh	decocted	19	
Lamiaceae	*Ocimum basilicum* L.	PA-46	albacahar	N	leaves	anxiety	F	fresh	raw	7	36.0
						bad wind	S	fresh	raw	3	
						**dizzy**	**Q**	fresh	infusion	9	
						evil eye	S	fresh	bath	4	
						high pressure	B	fresh	infusion	1	
						nausea	G	fresh	infusion	1	
Lamiaceae	Salvia spp.	PA-47	salvia	En	leaves	**spasm**	**E**	fresh	decocted	2	100.0
Lauraceae	*Cinnamomum verum* J.Presl	PA-48	mango con canela	Ex	leaves	abortive	**D**	fresh	decocted	2	58.3
						chinkunguya	P	fresh	decocted	2	
						cough	A	fresh	decocted	1	
						colic pain	**D**	fresh	decocted	5	
						drink	S	fresh	decocted	2	
Lauraceae	*Persea americana* Mill.	PA-49	aguacate	N	seed	abortive	D	fresh	decocted	5	86.2
Lauraceae	*Persea americana* Mill.		aguacate oloroso	N	leaves	**diarrhoea**	**G**	fresh	infusion	19	
					seed	kidney problem	C	fresh	liquefied	3	
					leaves	**nausea**	**G**	fresh	infusion	2	
					leaves	**stomach ache**	**G**	fresh	infusion	29	
Loranthaceae	*Struthanthus crassipes* (Oliver) Eichl.	PA-50	secapalo	En	leaves	**grains**	**O**	fresh	decocted	7	53.3
						kidney problem	C	fresh	decocted	7	
						**wounds**	**O**	fresh	bath	1	
Malvaceae	*Guazum aulmifolia* Lam.	PA-51	guazima	N	**bark**	**colitis**	**G**	dry	decocted	5	61.9
					bark	diabetes	H	dry	decocted	4	
					**bark**	**diarrhoea**	**G**	dry	decocted	4	
					fruit	drink	S	fresh	squeezed	2	
					**bark**	**stomach ache**	**G**	dry	decocted	4	
					leaves	veterinary	R	fresh	raw	2	
Malvaceae	*Heliocarpus appendiculatus* Turcz.	PA-52	jonote	N	latex	**wounds**	**O**	dry	topical raw	4	100.0
Malvaceae	*Sida rhombifolia* L.	PA-53	malva and albacahar	N	leaves	**bad wind**	**S**	fresh	raw	1	100.0
					stem	**ritual**	**S**	fresh	raw	4	
Malvaceae	*Sphaeralcea angustifolia* (Cav.) G.Don	PA-54	hierva del negro	N	whole plant	**bad wind**	**S**	fresh	raw	15	100.0
Meliaceae	*Azadirachta indica* A. Juss.	PA-55	neem	Ex	fruit	**diabetes**	**H**	fresh	infusion	18	100.0
Meliaceae	*Cedrela odorata* L.	PA-56	cedro	N	bark	**abortive**	**D**	dry	decocted	4	75.0
					bark	fever	P	dry	decocted	1	
					leaves	inflammation	E	fresh	decocted	1	
					bark	**problems in trying to have children**	**D**	dry	decocted	2	
Meliaceae	*Melia azedarach* L.	PA-57	piocha	Ex	leaves	diabetes	H	fresh	infusion	8	100.0
Monimiaceae	*Peumus boldus* Molina	PA-58	boldo	Ex	leaves	colitis	G	fresh	infusion	4	100.0
Moraceae	*Morus celtidifolia* Kunth	PA-59	mora	N	leaves	**chinkunguya**	**P**	fresh	decocted	2	66.7
					leaves	tooth pain	L	fresh	raw	1	
Moringaceae	*Moringa oleifera* Lam.	PA-60	moringa	Ex	leaves	cancer	I	fresh	decocted	1	85.7
					leaves	**diabetes**	**H**	fresh	decocted	6	
Musaceae	*Musa* spp.	PA-61	platano	Ex	bark	respiratory system	A	fresh	decocted	2	77.8
					bark	**tuberculosis**	**P**	fresh	fermented	7	
Myrsinaceae	*Ardisia compressa* Kunth	PA-62	capulin and nona	N	leaves	**stomach ache**	**G**	fresh	infusion	5	62.5
					leaves	wounds	O	fresh	bath	3	
Myrtaceae	*Eucalyptus globulus* Labill	PA-63	eucalipto	Ex	leaves	**bronchitis**	**A**	fresh	decocted	3	100.0
Myrtaceae	*Pimenta dioica*(L.) Merr.	PA-64	pimienta	Ex	leaves	**flu**	**A**	fresh	decocted	3	100.0
Myrtaceae	*Psidium guajava* L.	PA-65	guayaba	N	leaves	**diarrhoea**	**G**	dry	decocted	16	53.3
					bark	flu	A	dry	decocted	14	
Myrtaceae	*Syzygium aromaticum* (L.) Merr. & Perry	PA-66	clavo	Ex	seed	tooth pain	L	dry	topical raw	5	100.0
Nyctaginaceae	*Bougainville aglabra Choisy*	PA-67	bugambilia	Ex	flower	cough	A	fresh	infusion	19	100.0
Orchidaceae	*Vanilla planifolia* Jacks. ex Andrews	PA-68	vainilla	N	fruit	**drink**	**S**	fresh	fermented	2	72.7
					fruit	**drink**	**S**	fresh	raw	6	
					fruit	menopause	D	fresh	tincture	1	
					leaves	menopause	D	fresh	decocted	2	
Papaveraceae	*Fumaria officinalis* L.	PA-69	Sangre de Cristo (Blood of Christ)	Ex	leaves	wounds	O	fresh	bath	3	100.0
Passifloraceae	*Passiflora coriacea* Juss.	PA-70	hierva del murcielago	N	leaves	kidney problem	C	fresh	decocted	6	100.0
Pedaliaceae	*Sesamum indicum* L.	PA-71	ajonjoli	Ex	seed	breastfeeding	D	dry	decocted	15	100.0
Piperaceae	*Peperomia granulosa* Trel.	PA-72	gordonzillo (acoyo)	N	root	**breastfeeding**	**D**	fresh	decocted	5	53.1
					stems	**menstruation**	**D**	fresh	decocted	4	
					root	**to have children**	**D**	fresh	decocted	8	
					leaves	cirrhosis	N	fresh	infusion	1	
					leaves	rheumatism	E	fresh	burned	9	
			acoyo (gordonsillo) and ajo		leaves	respiratory system	A	fresh	infusion	5	
Piperaceae	*Piper sanctum* (Miq.) Schltdl. ex C.DC.	PA-73	hierva santa	N	leaves	clean baby and post-parthum	D	fresh	decocted	2	100.0
Plantaginaceae	*Plantago major* L.	PA-74	llanten	Ex	leaves	skin problems	O	fresh	decocted	15	100.0
Poaceae	*Cymbopogon citratu Cymbopogon citratus* (DC.) Stapf	PA-75	zacate limon	Ex-invader	leaves	drink	S	fresh		7	100.0
Poaceae	*Pachystachys spicata* (Ruiz & Pav.) Wassh.	PA-76	mohuite	N	stem	bad wind	S	fresh		1	70.6
					leaves	epilepsy	F	fresh	decocted	3	
						**kidney problem**	**C**	fresh	decocted	12	
						nausea	G	fresh	infusion	1	
Poaceae	*Phalaris canariensis* L.	PA-77	alpistle	Ex	seed	diabetes	H	fresh	liquefied	9	100.0
Poaceae	*Zea maiz* L.	PA-78	maiz morado	N	seed	alcoholic drink	S	fresh	fermented	3	82.4
			pelo de maiz		fruit	**kidney problem**	**C**	fresh	infusion	14	
Portulacaceae	*Portulaca oleraceae* L.	PA-79	verdolaga	N	leaves	blood circulation	B	fresh	burned	2	100.0
Rosaceae	*Eriobotrya japonica* (Thunb.) Lindl.	PA-80	nispero	Ex	leaves	kidney problem	C	fresh	decocted	2	100.0
Rosaceae	*Prunus domestica* L.	PA-81	ciruela	Ex	leaves	rash and grains	O	fresh	smashed	4	66.7
						smallpox	P	fresh	bath	2	
Rubiaceae	*Hamelia patens* Jacq.	PA-82	tres hojitas	N	leaves	anaemia	B	fresh	infusion	3	23.4
						blood circulation	B	fresh	decocted	7	
						breastfeeding	D	fresh	burned	6	
						cancer	I	dry	decocted	9	
						colitis	G	fresh	decocted	4	
						**diabetes**	**H**	fresh	decocted	7	
						**diabetes**	**H**	fresh	infusion	11	
						gastritis	G	fresh	infusion		
						gastritis	G	fresh	decocted	8	
						high pressure	B	fresh	infusion	1	
						menstruation	D	fresh	decocted	2	
					root	respiratory system	A	dry	decocted	7	
						skin problems, fungus	O	fresh	squeezed	2	
						ulcers	G	fresh	decocted	5	
						wounds	O	fresh	bath	5	
Rubiaceae	*Morinda citrifolia* L.	PA-83	noni	Ex	fruit	**diabetes**	**H**	fresh	liquefied	10	83.3
						heart problems	B	fresh	squeezed	2	
Rutaceae	*Casimiroa edulis* La Llave	PA-84	zapote blanco	N	leaves	**cholesterol**	**B**	fresh	infusion	3	30.0
					latex	gum	L	dry	raw	2	
					bark	diabetes	H	dry	decocted	1	
					leaves	fever	P	fresh	infusion	2	
					bark	kidney problem	C	dry	decocted	2	
Rutaceae	*Citrus aurantiifolia* (Christm.) Swingle	PA-85	azares de naranjo	Ex	leaves	**anxiety**	**F**	fresh	decocted	3	100.0
Rutaceae	*Citrus× aurantium* L.	PA-86	naranja cucha	Ex	leaves	**anxiety**	**F**	fresh	decocted	9	64.3
						cough	A	fresh	decocted	4	
			naranja, papaya, limon, nopal		fruit	diabetes	H	fresh	liquefied	1	
Rutaceae	*Citrus limetta* Risso	PA-87	lima chichi	Ex	fruit	high pressure	B	fresh	decocted	3	75.0
						**infection in the eyes**	**Q**	fresh	squeezed	9	
Rutaceae	*Citrus medica* L.	PA-88	limon	N	fruit	cough	A	fresh	infusion	14	
Rutaceae	Citrus sinensis (L.) Osbeck	PA-89	naranja	Ex	leaves	**anxiety**	**F**	fresh	infusion	6	54.5
						flu	A	fresh	infusion	5	
Rutaceae	*Murraya paniculata* (L.) Jack	PA-90	limonaria	Ex	leaves	**diabetes**	**H**	fresh	squeezed	2	66.7
						tooth pain	L	fresh	decocted	1	
Rutaceae	*Ruta graveolens* L.	PA-91	ruda	Ex	leaves	colitis	G	fresh	infusion	3	43.8
						evil eye	S	fresh	raw	3	
						gastritis	G	fresh	infusion	4	
						high pressure	B	fresh	infusion	4	
						**menstruation**	**D**	fresh	infusion	8	
						pain in ears	Q	fresh	infusion	3	
						**abortive**	**D**	fresh	infusion	6	
						pain in the chest	A	fresh	infusion	1	
Sapotaceae	*Manilkara chicle* (Pittier) Gilly	PA-92	zapote chico and guia del chayote	N	leaves	**cholesterol**	**B**	fresh	infusion	4	57.1
						diabetes	H	fresh	infusion	1	
						high pressure	B	fresh	infusion	2	
Sapotaceae	*Pouteria sapota* (Jacq.) H.E. Moore & Stearn.	PA-93	zapote rebentador	N	bark	**diabetes**	**H**	dry	decocted	4	57.1
					fruit	diarrhoea	G	fresh	squeezed	2	
					leaves	nausea	G	fresh	decocted	1	
Smilacaceae	*Smilax mollis* Humb. & Bonpl. ex Willd.	PA-94	cocolmecate (bejuco enredadera)	N	root	**diabetes**	**H**	dry	decocted	4	57.1
					root	gastritis	G	dry	decocted	1	
					bark	loss weight	B	dry	decocted	2	
Solanaceae	*Physalis ixocarpa* Brot. ex Hornem	PA-95	tomate verde	N	leaves	**kidney problem**	**C**	fresh	infusion	4	100.0
Urticaceae	*Cecropia obtusifolia* Bertol.	PA-96	hormiguillo (nihuiya)	N	bark	**diabetes**	**H**	dry	decocted	2	100.0
Verbenaceae	*Lippia duartei* Moldenke	PA-97	hierva dulce	N	whole plant	**diabetes**	**H**	dry	decocted	1	50.0
					leaves	diarrhoea	G	dry	decocted	1	
Verbenaceae	*Lippia graveolens* Kunth	PA-98	oregano	N	leaves	**respiratory system**	**A**	dry	infusion	4	100.0
Xanthorrhoeaceae	*Aloe vera* (L.) Burm.f.	PA-99	savila	Ex	leaves	gastritis	G	fresh	raw	7	33.3
						hair problems	Q	fresh	smashed	6	
						inflammation	E	fresh	topical raw	3	
					whole plant	ulcers	G	fresh	infusion	3	
						**wounds**	**O**	fresh	topical raw	11	
						anaemia	B	fresh	infusion	1	
						chinkunguya	P	fresh	infusion	2	
Zingiberaceae	*Costus spicatus* (Jacq.) Sw.	PA-100	caña de jabali	N	stem	**kidney problem**	**C**	dry	infusion	21	95.5
			caña de jabali con raiz de chiote		root	hepatitis	N	dry	decocted	1	
Zingiberaceae	*Zingiber officinale* Roscoe	PA-101	gengibre	Ex	root	anaemia	B	fresh	decocted	1	54.5
						**blood circulation**	**B**	fresh	decocted	2	
						**clean the blood**	**B**	fresh	raw	3	
						colic pain	D	fresh	decocted	1	
						stomach ache	G	fresh	decocted	1	
						intestinal worm	G	fresh	decocted	2	
						inflammation	E	fresh	decocted	1	

A, refers to Respiratory system disorders; B to Blood cardiovascular disorders; C, Kidney disorders; D, Genio-urinary disorders and childcare; E, Skeleton-muscular system disorders, F, Nervous system disorders; G, Gastro-intestinal disorders; H, Endocrinal disorders; I, Oncology; L, Dental care; M, Poisonous bites; N, Liver disorders; O, Skin disorders; P, Fever and infective diseases; Q, Ear, eye, hair disorders; R, Veterinary uses, S, Different uses. N, native, En, endemic, Ex, exotic; Bold ailments treated, Main use of Plant on which FL% is based.

**Table 3:** Number and percent of use reports (UR) and important consensus factors (ICF) of the Mexican plant species for each ailment category.

N°	AILMENT CATEGORIES	N° SPECIES	N° of UR	% UR	ICF
A	Respiratory system disorders	17	100	7.89	0.84
B	Blood-cardiovascular disorders	21	112	8.83	0.82
C	Kidney disorders	17	119	9.38	0.86
D	Genital-urinary disorders and childcare	17	118	9.31	0.86
E	Skeleton-muscular system disorders	8	34	2.68	0.79
F	Nervous system disorders	5	28	2.21	0.85
G	Gastro-intestinal disorders	29	247	19.48	0.89
H	Endocrinal disorders	23	137	10.80	0.84
I	Oncology	5	44	3.47	0.91
L	Dental care	9	27	2.13	0.69
M	Poisonous bites	2	14	1.10	0.92
N	Liver disorders	3	3	0.24	0.00
O	Skin disorders	17	83	6.55	0.80
P	Fever and infective diseases	13	93	7.33	0.87
Q	Ear-eye-hair disorders	7	39	3.08	0.84
R	Veterinary uses	4	14	1.10	0.77
S	Different uses	11	56	4.42	0.82

UR, Use-reports; ICF, Informant’s consensus factor.
